# Plant Growth Regulator- and Elicitor-Mediated Enhancement of Biomass and Andrographolide Production of Shoot Tip-Culture-Derived Plantlets of *Andrographis paniculata* (Burm.f.) Wall. (Hempedu Bumi)

**DOI:** 10.3390/plants12162953

**Published:** 2023-08-15

**Authors:** Aicah Patuhai, Puteri Edaroyati Megat Wahab, Martini Mohammad Yusoff, Yaser Hassan Dewir, Ali Alsughayyir, Mansor Hakiman

**Affiliations:** 1 Department of Crop Science, Faculty of Agriculture, Universiti Putra Malaysia (UPM), Serdang 43400, Selangor, Malaysia; aicahpatuhai@gmail.com (A.P.); putri@upm.edu.my (P.E.M.W.); martinimy@upm.edu.my (M.M.Y.); 2 Plant Production Department, College of Food and Agriculture Sciences, King Saud University, Riyadh 11451, Saudi Arabia; ydewir@ksu.edu.sa; 3 Department of Plant and Soil Sciences, Mississippi State University, 75 B.S. Hood Rd, Starkville, MS 39762, USA; aa2942@msstate.edu; 4 Laboratory of Sustainable Resources Management, Institute of Tropical Forestry and Forest Products, Universiti Putra Malaysia (UPM), Serdang 43400, Selangor, Malaysia

**Keywords:** chitosan, contamination, elicitation, plant growth regulators, salicylic acid, surface sterilization

## Abstract

*Andrographis paniculata* (Burm.f.) Wall. (Acanthaceae) is revered for its medicinal properties. In vitro culture of medicinal plants has assisted in improving both the quantity and quality of their yield. The current study investigated the effects of different surface sterilization treatments, plant growth regulators (PGRs), and elicitors on culture establishment and axillary shoot multiplication of *A. paniculata*. Subsequently, the production of andrographolide in the in vitro plantlets was evaluated using high-performance liquid chromatography (HPLC) analysis. The shoot-tip explant was successfully sterilized using 60% commercial bleach for 5 min of immersion with a 90% survival rate and 96.67% aseptic culture. The optimal PGR for shoot growth was 6-benzylaminopurine (BAP) at 17.76 µM, supplemented into Murashige and Skoog (MS) media, producing 23.57 ± 0.48 leaves, 7.33 ± 0.10 shoots, and a 3.06 ± 0.02 cm length of shoots. Subsequently, MS medium supplemented with 5 mg/L chitosan produced 26.07 ± 0.14 leaves, 8.33 ± 0.07 shoots, and a 3.63 ± 0.02 cm length of shoots. The highest andrographolide content was obtained using the plantlets harvested from 5 mg/L chitosan with 2463.03 ± 0.398 µg/mL compared to the control (without elicitation) with 256.73 ± 0.341 µg/mL (859.39% increase). The results imply that the protocol for the shoot-tip culture of *A. paniculata* was developed, and that elicitation enhanced the herbage yield and the production of andrographolide.

## 1. Introduction

*Andrographis paniculata* (Burm.f.) Wall., commonly known as the King of Bitters, belongs to the family Acanthaceae. It is widely used in East Asian, South Asian, and Southeast Asian traditional remedies. Leaves and aerial parts of this plant are usually employed in traditional medicine to treat hepatitis, bronchitis, colitis, cough, fever, mouth ulcers, sores, tuberculosis, bacillary dysentery, venomous snake bites, common cold, urinary tract infections, and diarrhea [1]. Lactone terpenoids, specifically andrographolide, dehydroandrographolide, neoandrographolide, and deoxyandrographolide, are the main bioactive compounds produced in *A. paniculata* [1]. Andrographolide is a promising candidate to remediate various diseases such as inflammation, colds, and cancer [2].

The initial step of in vitro culture is the obtaining of microorganism-free explants from the mother plant to be propagated by tissue culture. The contamination problem is the critical limiting factor that is frequently encountered at the early stage. The type of sterilization agent, its concentrations, and the time of immersion are all factors to consider during the sterilization process [3,4]. The most often-used sterilization agents include sodium hypochlorite (NaClO), mercuric chloride (HgCl_2_), hydrogen peroxide (H_2_O_2_), ethanol (C_2_H_5_OH), and silver nitrate (AgNO_3_). However, Suraya et al. [5] found that mercury chloride can be hazardous due to its volatile properties at room temperature and was excessively toxic to humans and the environment.

Biotechnological approaches, particularly those involving plant tissue culture, help produce desirable bioactive metabolites [6]. Higher production of bioactive and secondary metabolites has been reported in many tissue-cultured herbs compared to their mother plants. Plant tissue culture offers an alternative method that can overcome the limitations of extracting valuable bioactive and secondary metabolites, limits which arise from constrained natural resources [5].

Axillary shoot multiplication effectively addresses the problems related to conventional propagation techniques, such as limited seed viability and slow growth rate [7]. A previous study found that direct shoot regeneration from the nodal segment, seed and leaf-derived callus, and stem of *A. paniculata* was achieved when cultured on media supplemented with BAP and 1-naphthalene acetic acid (NAA) [8]. Another study reported that the multiplication of *Andrographis paniculata* shoots can be achieved using BAP with indole-3-acetic acid (IAA) [9]. Variables such as growth regulators, nutrient media, and light conditions can be optimized to enhance the biomass production and secondary metabolites of *A. paniculata* [10].

Elicitation using microbial, physical, and chemical agents is a practice commonly used to regulate plant growth and induce stress in the culture. Chitosan has also been reported to have enhanced natural defense responses in plants and has been used as a natural compound to control pre- and post-harvest pathogenic diseases [11]. Antimicrobial activities of chitosan-treated cultures against various phytopathogens have been reported [12]. Other than chitosan, salicylic acid (SA) can also enhance the bioactive metabolites from medicinal plants, which function in plant development and metabolism, crop production, and pest control [2]. Due to its hormone-like activity, SA has also synthesized and accumulated bioactive metabolites in various plant species via in vitro systems [6].

Enhancing bioactive metabolites through in vitro culture techniques is advantageous for developing models with scale-up potential. Hence, it can be widely employed as a novel technique to produce the desired bioactive metabolites [13]. The present study aimed to develop a reliable protocol for shoot-tip culture of *A. paniculata.* Surface sterilization, multiplication, elicitation, and the quantification of andrographolide production in in vitro plantlets of *A. paniculata* were conducted.

## 2. Results

### 2.1. Surface Sterilization

**Figure 1:**
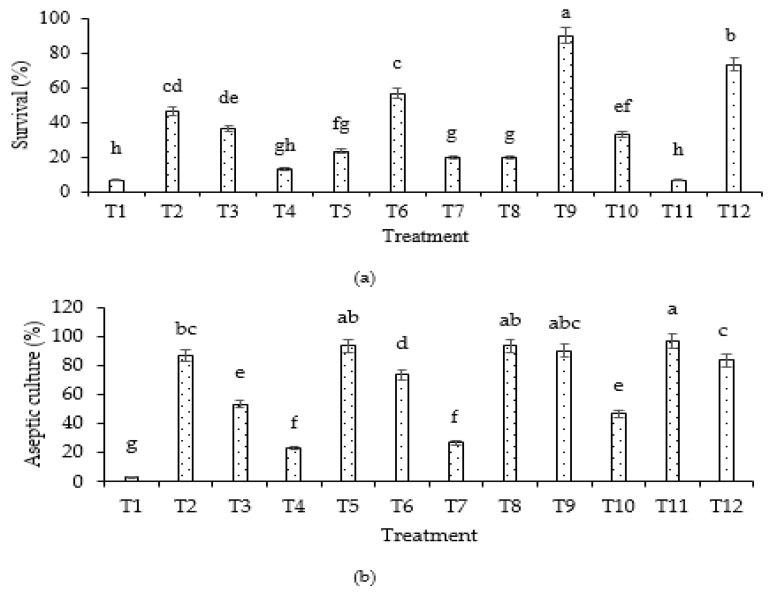
Effects of different commercial bleach concentrations and immersion durations on establishing sterile culture from shoot-tip explant of *A. paniculata*: (**a**) survival percentage; (**b**) aseptic culture percentage. T1 (40%, 1 min), T2 (40%, 10 min), T3 (40%, 5 min), T4 (50%, 1 min), T5 (50%, 10 min), T6 (50%, 5 min), T7 (60%, 1 min), T8 (60%, 10 min), T9 (60%, 5 min), T10 (70%, 1 min), T11 (70%, 10 min) and T12 (70%, 5 min), where values in percentage refer to commercial bleach concentration. Values are means of *n* = 30. T = treatment. Means followed by the same letters indicate that there is no significant difference (*p* < 0.05) using least significant means (LS-means).

The results showed that different concentrations and immersion durations of commercial bleach affected the survival percentage of the explants (Figure 1a). The highest survival percentage (90%) was observed in Treatment 9 (60% commercial bleach; 5 min immersion), while the lowest survival percentage was recorded in Treatments 1 and 11 with 40% and 70% commercial bleach with immersion for 1 and 10 min, respectively, with 6.67% survival. On the other hand, based on the obtained results, Treatment 11 (70% of commercial bleach; 10 min immersion) showed the highest aseptic culture percentage (96.67%) (Figure 1b), while the lowest aseptic culture percentage was recorded in Treatment 1 (40% of commercial bleach; 1 min immersion), with 3.33% of aseptic culture.

### 2.2. Shoot-tip Culture

**Figure 2:**
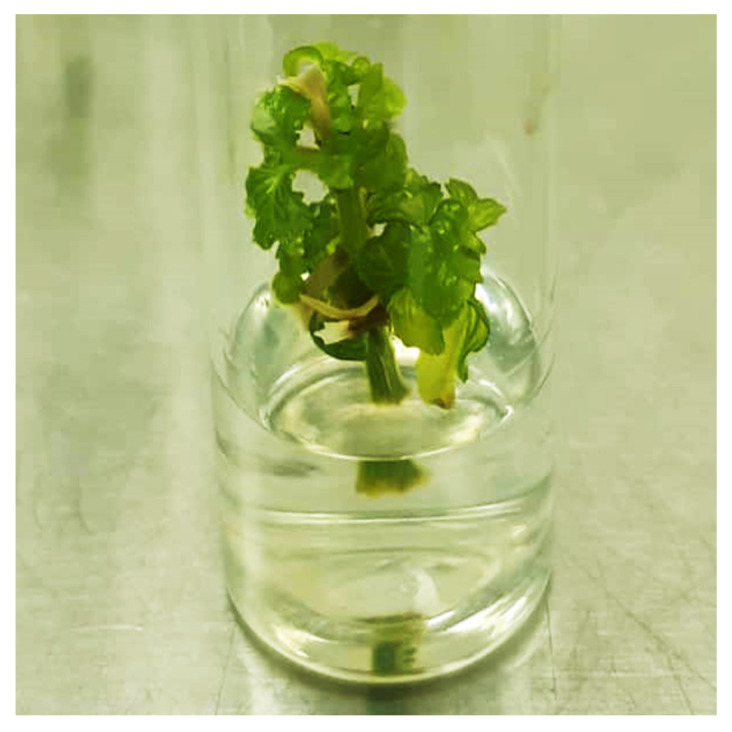
Shoot-tip culture of *A. paniculata* cultured on MS media supplemented with 17.76 µM BAP on day 21. BAP: 6-benzylaminopurine.

All the treatments of different PGRs, including the control, showed positive responses in the proliferation of buds when shoot-tip was used as explant. Parameters of the number of leaves, number of shoots, and length of shoots were increased from Treatments 1 to 6. The highest herbage yield for all parameters was observed in Treatment 6 (17.76 µM BAP), with 23.57 ± 0.48 leaves, 7.33 ± 0.10 shoots, and a 3.06 ± 0.02 cm length of shoot (Table 1, Figure 2), while Treatment 1 (control) showed the lowest herbage yield with 5.03 ± 0.08 leaves, 1.70 ± 0.04 shoots and a 2.25 ± 0.02 cm length of shoot, respectively.

### 2.3. Elicitation

From the previous experiment, an *A. paniculata* explant was excised from plantlets cultured on optimal medium for shoot-tip culture (MS + 17.76 µM BAP). MS medium supplemented with 17.76 µM BAP was subjected to different concentrations of chitosan and SA (1–5 mg/L, with 1 mg/L interval). The effect of the different chitosan- and SA-treated cultures were assessed in terms of the numbers of leaves and shoots, and the length of the shoots. The results showed an incremental trend in the parameters obtained when applied with different elicitors. The highest number of leaves and shoots and the highest length of shoots were recorded in MS media supplemented with 5.0 mg/L chitosan with 26.07 ± 0.14 leaves, 8.33 ± 0.07 shoots, and a 3.63 ± 0.02 cm length of shoots, respectively (Table 2). At the same time, the control treatment showed the lowest herbage yield with 23.57 ± 0.48 leaves, 7.33 ± 0.07 shoots, and a 3.06 ± 0.02 cm length of shoot, respectively. The same incremental trend could be observed when the shoot-tip explant was cultured onto MS media supplemented with SA until 4.0 mg/L SA, and then the trend starts to decline at the 5.0 mg/L SA treatment. This suggests that MS media supplemented with 4.0 mg/L SA showed the optimum treatment for SA-treated cultures, with 25.97 ± 0.06 leaves, 7.97 ± 0.10 shoots, and a 3.19 ± 0.00 cm length of shoot, respectively.

### 2.4. Quantification of Andrographolide in Shoot-tip Extracts of Micropropagated A. paniculata

**Figure 3:**
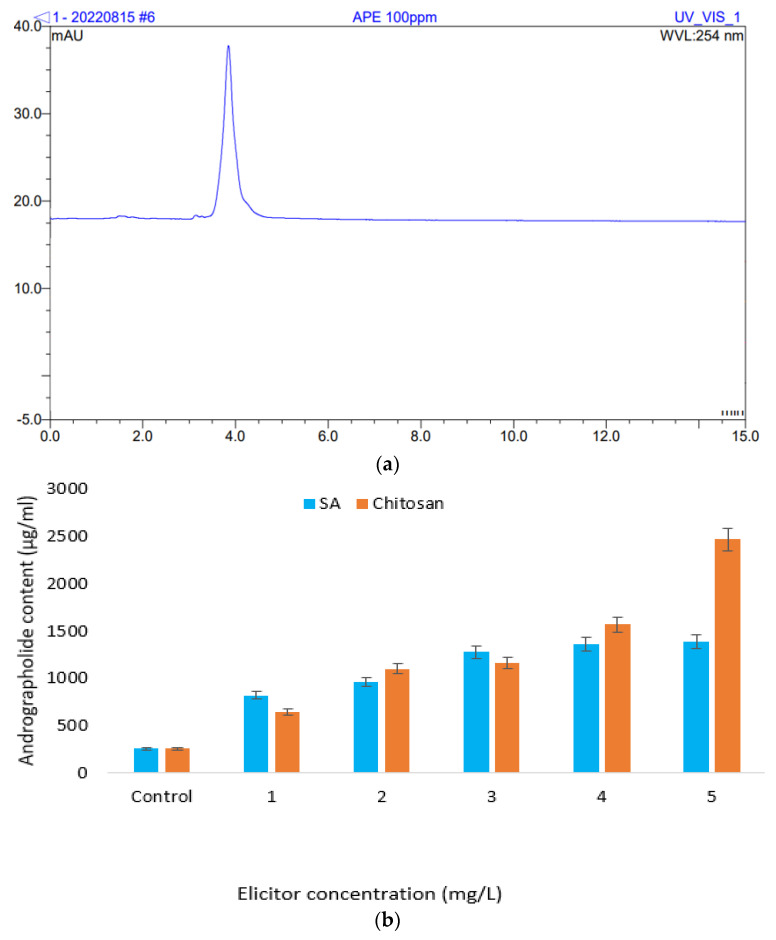
(**a**) Chromatogram of standard andrographolide; retention time 3.84 min at 254 nm and (**b**) andrographolide production of plantlets derived from shoot-tip culture under the influence of chitosan and salicylic acid at different concentrations.

The present study investigates andrographolide production from plantlets obtained from shoot-tip explants cultured onto MS media supplemented with chitosan and SA. A total of 5 mg of standard andrographolide was dissolved in 5 mL of 100% methanol (HPLC grade) obtaining a stock of 1000 µg/mL. The stock solution was further diluted to obtain the dilution range of the calibration curve. The range of the calibration curve of andrographolide standards was 0, 20, 40, 60 and 100 ppm, with a correlation coefficient of 0.9989. This indicated that the linearity of the studied method complied with the regulatory requirement. Figure 3a shows the chromatogram standard at 100 ppm. The highest andrographolide production was found in MS media treated with 5.0 mg/L chitosan (2463.03 ± 0.398 µg/mL), while the lowest andrographolide production was obtained from the control treatment, with 256.73 ± 0.341 µg/mL (Figure 3b) at retention time 3.84 min and 254 nm.

## 3. Discussion

Surface sterilization is used to achieve sterility in order to prevent bacterial and fungal contamination. The surface sterilization protocol increased the aseptic percentage in the shoot-tip explants. However, it also led to necrosis of the explants, thus making it harder to rejuvenate them [14,15,16]. Contamination in cultured explants can occur within three to ten days after inoculation, as mentioned by Chen and David [17]. However, if the contamination occurs at a later stage, it can be concluded that the plant materials used might be infected. Hence, regardless of how optimal and effective the surface sterilization protocol is, contamination will occur as a result of exudation from the endogenous microbial culture of the planting materials. Although antibiotics can be used to remediate the problem, it is advisable to select a healthy mother plant rather than treat them with antibiotics, which would incur more costs. Clorox, a bleach containing 5.25% NaClO, is an effective sterilizing agent for various plant species, but their responses can vary, depending on explant types. Different species and plant parts can produce a variety of observations. Herbaceous plants are more easily surface sterilized and produce more positive responses as compared to woody plants due to the juvenility of the plant tissues. Different plant parts also need to be considered when choosing the concentration of the sterilant. Plant parts such as stems and embryos encapsulated in a seed pod will not become necrotic as frequently as would young shoots or petals when used as explants for surface sterilization, as these tissues are more fragile. As observed in many studies, different explant types have different concentration requirements for sterilants, including *Phyllanthus niruri*, *Gerbera hybrida*, *Aquilaria malaccensis*, and *Zingiber officinale* [5,18,19,20]. The concentration of commercial bleach affects its effectiveness in reducing contaminants and its potential to cause tissue injury. A higher concentration of bleach reduces contaminants but can cause tissue injury, resulting in browning, low percentages of explant survival, and the death of explants. Prolonged exposure to high concentrations of sterilants will produce phenolics, which will also lead to the browning and death of explants. Hence, a balance between commercial bleach concentration and the duration of immersion needs to be considered when conducting surface sterilization. Browning can be prevented by reducing sterilant exposure, increasing NaClO concentration, or keeping cultures in dark conditions. The browning of explants may also result from the stress conditions induced by the in vitro system, as observed in herbs such as *Cestrum nocturnum* [21], *Solanum tuberosum* [22], *Zingiber officinale* [23] and *Matricaria chamomilla* [1].

Axillary bud proliferation is one of the micropropagation pathways that utilize axil parts (e.g., meristem, shoot-tip, and nodal segment) to develop into plantlets [24,25]. Since this technology does not involve cell dedifferentiation of differentiated cells but rather the development and growth of new shoots from pre-existing meristems, it has usually been pointed out as the most commercially viable way of propagating plants in vitro [26]. Among the methods utilizing axils, shoot-tip culture is the most widely used and considered the most commercially viable method for guaranteeing the genetic stability of the plantlets obtained [25]. Not only that, but shoot-tip culture is also a rapid technique that does not require special equipment in the manner of meristem tip culture, in which the meristem part needs to be isolated under the microscope. The application of cytokinin is essential, as it helps to break the bud dormancy phase of the explants. Cytokinins control the size of the shoot meristem, the number of leaf primordia, and the growth of the leaf and shoot by promoting cell division [25]. Hence, it is important to choose juvenile plant tissues due to the abundance of natural phytohormones, compared to plant tissues that already aging. 6-benzylaminopurine (BAP) is more effective for shoot proliferation than are other cytokinins (kinetin, thidiazuron, picloram) [27,28]. In the present study, all shoot-tip explants showed positive growth. However, the growth was slower in treatments without PGRs. This observation concurred with the understanding of the roles of cytokinin in plant growth by stimulating cell division. According to Nor Mayati and Jamnah [29], slow growth is probably due to insufficient nutrients before the differentiation stage. These results differed from the findings by Suraya et al. [5], who stated that aseptic nodal segments of *Phyllanthus niruri* cultured on MS medium fortified with 1.0 mg/L BAP produced the highest number of shoots and the combination of kinetin and BAP induced multiple shoot formation in all nodal explants. MS medium supplemented with high BAP produced better results for *Dracocephalum kotschyi* shoot propagation [30]. Similar results were achieved for the bud proliferation of *Musa acuminata* [31], *Mentha piperita* [27] and *Scutellaria altissima* [6].

It was known that the explants’ responses were different depending on the elicitors’ types. The elicitors play a vital role in maintaining organisms’ growth, but they are harmful to plants at high concentrations, as elicitors can act as stress agents to plants. When plants are under stress conditions, the herbage yield, biomass, and subsequently bioactive compounds will be altered. Using this knowledge, a number of research efforts have been conducted to enhance the herbage yield, biomass, and bioactive compounds in the treated plants. The increased biomass accumulation following chitosan application is due to the ability to boost the availability and absorption of water and essential nutrients by controlling the cells’ osmotic pressure [32]. A similar observation of biomass production has been reported for various in vitro culture systems for different plant species, such as callus culture of *Fagonia indica* [13], as well as cell suspension of *Silybum marianum* [32]. Chitosan has been reported to have a symbiotic relationship with growth-promoting rhizobacteria, thus triggering the germination rate and improving plant nutrient uptake [33]. The application of chitosan for herbage yields can be observed in many species, including *Artemisia aucheri* [34], safflower [6], *Swertia paniculata* [35], and *Coffea arabica* [36]. Different SA concentrations promote or inhibit plant growth in different plant species by modulating cell division and expansion [2]. SA regulates plant growth via multiple pathways [37]. The altered endogenous SA levels in plants can result in abnormal growth phenotypes [38]. Further investigation by Wang et al. [2] found that SA accumulation in the cad1 mutant promotes the quiescent center of cell division through the accumulation of reactive oxygen species and downregulation of the transcription factor genes. The results of the current study on SA application align with a previous study reported by Golkar et al. [6] and Koo et al. [38], in which plant growth was inhibited by applying high levels of elicitors. The reduction of callus production after adding high amounts of SA might be attributed to the stress induced upon cell growth and cell division. Previous studies’ results have shown the inhibitory effects of SA on plant growth development [39].

Plants are good sources for the discovery of pharmaceutical compounds and medicines. Natural products could be potential drugs for humans or livestock species, and these products and their analogs can act as intermediates for synthesizing useful drugs [40]. Phytochemical screening of medicinal plants is crucial in identifying new sources of therapeutically and industrially essential compounds [41,42]. In the current study, the production of andrographolide was higher when the MS media was supplemented with chitosan than it was with supplementation with SA. The andrographolide production was double when chitosan was applied in the MS media. The content of andrographolide and other diterpene lactones was higher than that reported by Jindal et al. [43] and Pawar et al. [44] when both studied different PGR combinations in the media. Another study on *Vitis vinifera* extract found that chitosan significantly enhanced the targeted bioactive compounds [45]. Moreover, the synthesis of polyphenolics, secoiridoid, glycosides, lignin, flavonoids, and phytoalexins was observed in *Fagonia indica* [13], *Swertia paniculata* [35] and *Silybum marianum* [32] after being treated with chitosan. SA-treated culture proved to increase the production of fatty acids from *Jatropha curcas* callus grown in vitro [46]. Additionally, SA regulates the production of swertiamarin and amarogentin glycosides in *Swertia paniculata* [35], and phenolic and flavonoid compounds in *Fagonia indica* [13]. Moreover, SA also acts as a self-protective agent in in vitro culture of *Nicotiana tabacum* [38] and *Arabidopsis thaliana* [47].

## 4. Materials and Methods

### 4.1. Plant Material

*A. paniculata* were collected from the nursery site at the Faculty of Agriculture, Universiti Putra Malaysia Serdang, Selangor. The plant sample was identified at the Biodiversity Unit, Institute of Bioscience, UPM, with reference number KM 0020/22.

### 4.2. Explant Surface Sterilization

The shoots of *A. paniculata* were chosen as explants in this experiment. The explant was prewashed with two drops of detergent for 15 min under tap water. The surface sterilization procedure was conducted under a laminar flow hood. The explants were sterilized for 1, 5, and 10 min of exposure using different concentrations (40, 50, 60, and 70%) of commercial bleach containing 5.2% sodium hypochlorite with one to two drops of Tween 20 as a wetting agent. After that, all the treatment solutions of T1 (40%, 1 min), T2 (40%, 10 min), T3 (40%, 5 min), T4 (50%, 1 min), T5 (50%, 10 min), T6 (50%, 5 min), T7 (60%, 1 min), T8 (60%, 10 min), T9 (60%, 5 min), T10 (70%, 1 min), T11 (70%, 10 min) and T12 (70%, 5 min) were discarded and the explants were rinsed at least three times with sterile distilled water.

### 4.3. Axillary Shoot Multiplication

Murashige and Skoog [48] (MS) medium supplemented with different concentrations of T1 (control), T2 (2.22 µM BAP + 0.49 µM IBA), T3 (2.22 µM BAP + 5.37 µM NAA), T4 (8.88 µM BAP + 2.69 µM NAA), T5 (8.88 µM BAP) and T6 (17.76 µM BAP) was mixed with 0.1 g/L of myo-inositol and 20 g/L of sucrose. A total of 3 g/L of Gelrite^®^ as a gelling agent was added and stirred until completely dissolved before the pH was adjusted to pH 5.6–5.8. The solution was heated in the microwave before being placed in vials and covered with aluminum foil. All of the labeled vials were placed in an autoclave and sterilized at 121 °C and 1.05 kg/cm² for 20 min.

### 4.4. Elicitation

Chitosan (C_56_H_103_N_9_O_39_) and salicylic acid (SA) (Sigma-Aldrich, St. Louis, MO, USA) were used for elicitation. Different concentration levels (0, 1.0, 2.0, 3.0, 4.0, and 5.0 mg/L) of both chitosan and SA were introduced to the MS supplemented with 17.76 µM BAP.

### 4.5. Growing Conditions

Vials (25 × 95 mm; Phytotech^®^, Lenexa, KS, USA) containing cultured explants were incubated for four weeks at 25 ± 3 °C under a photoperiod of 16 h light and 8 h darkness supplied by white fluorescent light (45 µmol/m²/s) in the incubation room.

### 4.6. Extraction of Andrographolide

Elicitated plantlets were harvested in the fourth week after inoculation. A total of 10 mg dried plantlets were powdered and dissolved in 5 mL of 100% methanol (HPLC grade). The sample was then extracted via sonication-assisted extraction (Fisherbrand^®^ FB155055, Waltham, MA, USA) (40 °C for 30 min) and quantified for andrographolide content. Each extract was mixed using a vortex (ZX3 Advanced Vortex Mixer) for 3 min and filtered through 0.45 µm nylon syringe membranes (Macherey Nagel, Hoerdt, France), and stored at −4 °C before HPLC analysis.

### 4.7. Chromatographic Parameters

Quantitative analysis of andrographolide of *A. paniculata* was carried out as per the protocol of Masaenah et al. [49] with minor modification using HPLC (Thermo ScientificTM DionexTM UltiMate 3000 UHPLC, Waltham, MA, USA). Chromatographic separation was performed using XBridge^®^ (Waters, Milford, MA, USA) C18 column (5 µm, 4.6 mm × 250 mm). The mobile phase consisted of a mixture of methanol and deionized water (50:50) and a 1 mL/min flow rate. The injection volume was 10 μL. The temperature of the column was controlled at 25 °C, and samples were detected using a PD-M20A photodiode array detector. The andrographolide (Merck Chemical, Saint-Quentin Fallavier, France) was identified by comparing the retention time of samples with reliable standard chromatographic peaks at 254 nm, and the andrographolide content was expressed as µg/mL.

### 4.8. Analysis of Data

The experiment was conducted in a completely randomized design and analyzed using one-way analysis of variance (ANOVA) through SAS software, version 9.4 (SAS Institute, Cary, NC, USA). Means comparisons were separated by the least significant mean (LS mean) and Tukey honestly significant difference (HSD) test at *p* < 0.05.

## 5. Conclusions

An in vitro culture of *A. paniculata* was successfully established using shoot-tip explants. BAP at 17.76 µM proved optimal for axillary shoot multiplication and growth. Chitosan at 5 mg/L further enhanced shoot multiplication and growth with 26.07 leaves, 8.33 shoots, and 3.63 cm of shoot-length per explant. An increase in andrographolide production was obtained in chitosan and SA-treated cultures. However, MS media supplemented with 5 mg/L chitosan produced higher andrographolide with 2463.034 ± 0.398 µg/mL, compared to that of the control with 256.73 ± 0.341 µg/mL.

## Figures and Tables

**Table 1:** Effects of plant growth regulators and their concentrations on the number of leaves, shoots, and lengths of shoot from shoot-tip explants of *A. paniculata*.

Treatment	Number of Leaves	Number of Shoots	Length of Shoot (cm)
T1 (control)	5.03 ± 0.08 e	1.70 ± 0.04 d	2.25 ± 0.02 e
T2 (2.22 µM BAP + 0.49 µM IBA)	8.43 ± 0.17 d	2.00 ± 0.05 c	2.46 ± 0.01 d
T3 (2.22 µM BAP) + 5.37 µM NAA)	8.40 ± 0.18 d	1.80 ± 0.05 cd	2.28 ± 0.01 e
T4 (8.88 µM BAP + 2.69 µM NAA)	18.30 ± 0.21 c	3.60 ± 0.05 b	2.71 ± 0.02 c
T5 (8.88 µM BAP)	19.70 ± 0.38 b	3.8 ± 0.05 b	2.82 ± 0.01 b
T6 (17.76 µM BAP)	23.57 ± 0.48 a	7.33 ± 0.10 a	3.06 ± 0.02 a

Values are means ± standard error (SE) of *n* = 30. Means followed by the same letters within the same column indicate no significant difference (*p* < 0.05) using Tukey’s honestly significant difference (HSD) analysis. BAP: 6-benzylaminopurine; IBA: indole-3-butyric acid; NAA: 1-naphthalene acetic acid.

**Table 2:** The effect of different elicitors and their concentrations on the number of leaves, number of shoots, and the length of shoots (cm) using shoot-tip explant of *A. paniculata*.

Treatment (mg/L)	Number of Leaves	Number of Shoots	Length of Shoots (cm)
Control	23.57 ± 0.48 b	7.33 ± 0.07 c	3.06 ± 0.02 de
1.0 SA	23.60 ± 0.31 b	7.37 ± 0.10 c	2.69 ± 0.02 h
2.0 SA	23.63 ± 0.24 b	7.47 ± 0.04 c	2.88 ± 0.02 g
3.0 SA	24.70 ± 0.33 ab	7.53 ± 0.04 c	2.93 ± 0.02 gf
4.0 SA	25.97 ± 0.06 a	7.97 ± 0.10 b	3.19 ± 0.00 c
5.0 SA	24.77 ± 0.13 ab	7.90 ± 0.04 b	2.98 ± 0.01 ef
1.0 Chitosan	23.80 ± 0.50 b	7.37 ± 0.06 c	3.09 ± 0.02 d
2.0 Chitosan	24.03 ± 0.37 b	7.57 ± 0.03 c	3.10 ± 0.02 d
3.0 Chitosan	24.17 ± 0.30 b	7.90 ± 0.04 b	3.33 ± 0.02 b
4.0 Chitosan	24.53 ± 0.08 b	7.93 ± 0.04 b	3.34 ± 0.02 b
5.0 Chitosan	26.07 ± 0.14 a	8.33 ± 0.07 a	3.63 ± 0.02 a

Note: Values are means ± standard error (SE) of *n* = 30. Means followed by the same letters within the same column indicate that there is no significant difference (*p* < 0.05) using Tukey’s honestly significant difference (HSD) analysis. SA: salicylic acid.

## Data Availability

All data are presented in the article.
